# Changes in Metabolites Produced in Wheat Plants Against Water-Deficit Stress

**DOI:** 10.3390/plants14010010

**Published:** 2024-12-24

**Authors:** Valentina Spanic, Jurica Duvnjak, Dubravka Hefer, John C. D’Auria

**Affiliations:** 1 Agricultural Institute Osijek, Južno Predgrađe 17, 31000 Osijek, Croatia; jurica.duvnjak@poljinos.hr (J.D.); dubravka.hefer@poljinos.hr (D.H.); 2 Leibniz Institute of Plant Genetics and Crop Plant Research (IPK Gatersleben), OT Gatersleben, Corrensstraße 3, 06466 Seeland, Germany; dauria@ipk-gatersleben.de

**Keywords:** metabolic profiling, GC-MS, winter wheat, abiotic stress, drought stress

## Abstract

Drought stress can adversely affect the seed germination and seedling growth of wheat plants. This study analyzed the effect of drought on seed germination and the morphological parameters of seedlings from ten winter wheat genotypes. The primary focus was to elucidate the effects of two drought intensities on metabolic status in wheat seedlings. The findings suggest that most wheat genotypes exhibited a significant reduction in germination and growth traits under severe drought, while the genotype Srpanjka exhibited less reduction under both drought conditions. Out of 668 metabolic features, 54 were altered under 10% PEG stress and 140 under 20% PEG stress, with 48 commonly shared between these two stress intensities. This study demonstrated that the metabolic response of shoots to 10% PEG stress contrasts with that of 20% PEG stress. Some growth metabolites, such as oxalic acid, sophorose, and turanose, showed the highest positive increase under both stresses, while butanoic acid, tropic acid, glycine, propionic acid, and phosphonoacetic acid decreased. It is suggested that the accumulation of amino acids, such as proline, contributed to the drought tolerance of the plants. Among all organic acids, succinic and aspartic acids particularly increased the plant response to mild and severe drought stress, respectively. Our results suggest that different metabolites in wheat seedlings enhance the potential ability of wheat to cope with drought stress in the early growth stages by activating a rapid and comprehensive tolerance mechanism. This discovery presents a new approach for enhancing wheat tolerance to abiotic stress, including water deficit.

## 1. Introduction

Throughout the vegetative season, plants are exposed to various abiotic and biotic stresses. Abiotic stress, including drought, is one of the most significant problems affecting plant growth and development [1] and plant productivity [2]. Climate changes are increasing the average global temperature and leading to more frequent high-temperature extremes, such as heatwaves [3]. Alarmingly, it is predicted that up to 60% of the current wheat-growing area will be affected by severe water scarcity events by the end of the twenty-first century [4]. Therefore, drought-induced limitations on grain yields, causing economic losses, pose a threat to global food security. As the world population is projected to grow to an estimated 8.5 billion by 2030, it is essential to increase the grain yield of crops to meet the growing food demand [5]. Wheat (*Triticum aestivum* L.), one of the most important cereals, continues to play a crucial role in ensuring global food and nutrition security. Due to its widespread use in different regions, wheat frequently confronts drought stress as a result of climate changes. Developing and producing drought-tolerant wheat varieties with greater water-use efficiency is crucial, especially in the context of food sustainability [6]. Furthermore, it is expected that more drought-tolerant plants would have a stronger ability to initiate defense mechanisms against water deficit.

Drought can damage wheat plants at any point during the growing season, but specific stages, such as seedlings, are particularly influential in the further development of plants [7]. The first step in successful wheat production requires well-developed seedlings with a viable plant from each seed. Therefore, seed germination and seedling establishment are potentially the most critical stages in the life cycle of plants under drought conditions [8]. These two stages of wheat growth are significantly influenced by temperature and moisture conditions [9]. Water deficiency can greatly affect seed germination and early seedling growth by inducing oxidative damage and metabolic disruptions in plant cells [10]. Seeds lose vigor and become increasingly sensitive to stress upon germination, which occurs between imbibition and the emergence of the radicle from the seed [11]. Additionally, seeds are more tolerant to environmental stressors than seedlings. However, drought-induced reductions in traits and final grain yield depend on the severity and duration of the stress period. It is also important to note that wheat genotypes exhibiting drought tolerance at the germination stage tend to maintain this tolerance in later growth stages [12]. Previously, there was research conducted on after anthesis water deficit where it was evident that significant changes occurred at the metabolomic level in developing grains during the time from anthesis to maturity [13].

Drought stress causes morphological, physiological, biochemical, and molecular changes in plants as they attempt to mitigate the adversities of abiotic stresses. These changes, along with altered metabolic processes, result in a significant reduction in grain yield [14]. Kumar et al. [15] concluded that metabolic regulation is the key mechanism for maintaining cell osmotic potential under drought stress, as metabolites play a crucial role in plant growth and development through cell integrity, energy storage, cell signaling, membrane formation and scaffolding, and whole-plant resource allocation [16]. Thus, plants can modify their physiological mechanisms under drought stress to adapt through metabolic homeostasis. Metabolomics, transcriptomics, proteomics, and ionomics provide a broader scope of mechanisms underlying biological processes that can contribute to the identification and characterization of genes, proteins, metabolites, and ions involved in signaling pathways under drought stress [17]. Consequently, important metabolic pathways such as photosynthesis, sugar synthesis, the tricarboxylic acid cycle, glycolysis, and hormone synthesis are further affected [18]. Limited water availability and reduced CO_2_ assimilation result in disturbances in photosynthesis in chloroplasts, leading to excessive generation of reactive oxygen species that disrupt various metabolic pathways. The accumulation of osmolytes and osmoprotectants affects both primary and secondary metabolism [4]. In wheat, increased concentrations of metabolites such as tricarboxylic acid intermediates, sugars, and amino acids have been implicated in response to drought [19]. Further, Saeidi et al. [13] reported that abscisic acid (ABA) and sucrose concentration and sucrose synthase activity were significantly higher 17 days after anthesis than 10 days after anthesis during drought in developing grains. It is also important to note that in drought outlearn varieties, the sum of the amino acid concentrations determined, i.e., asparagine (22%), aspartic acid (48%), glycine (48%), ACC (79%), and valine (133%), were significantly increased. Other research showed that drought implemented 22 days after sowing resulted in an increase in metabolites, such as some amino acids, most notably proline, some organic acids, and lipid classes PC 36:3 and TAG 56:9 [19]. In flag leaves of wheat varieties, it was found that the application of salicylic acid, trehalose, or their interaction induced a marked increase in growth vigor of root and shoot, water relations, and protein, as well as nucleic acids in flag leaves [20]. However, many aspects of metabolites in plant drought tolerance at different growth stages of wheat remain to be revealed, especially in seedlings stage when our research was conducted. Thus, the main objectives of the present study were to compare metabolic changes in wheat seedlings subjected to drought stress during germination and the seedling stage. Additionally, we aimed to identify the most important metabolites associated with drought stress.

## 2. Results

### 2.1. Morphological Effects of Two Drought Stress Treatments in Wheat

**Figure 1:**
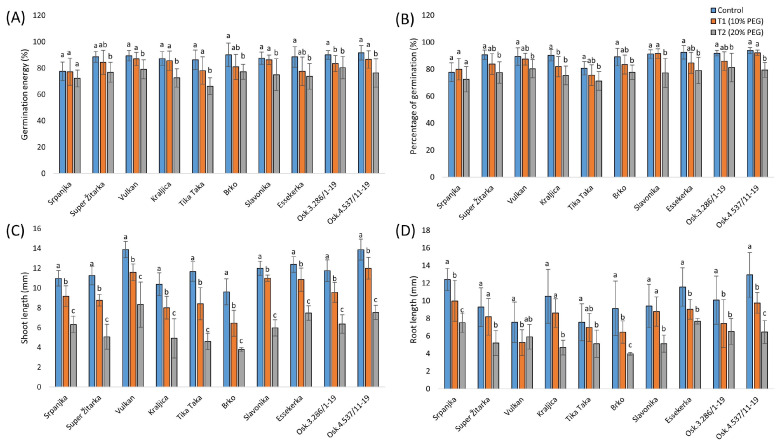
Germination energy (**A**), percentage of germination (**B**), shoot length (**C**), and root length (**D**) of ten winter wheat genotypes under control and two polyethylene glycol (PEG) treatments (T1-10% and T2-20%). Data are average values of ten biological replicates ± SD. Significant differences among treatments, for each genotype, separately, were assessed by the Fisher LSD test. Trait means with the same letter do not significantly differ at *p* < 0.05.

The seeds treated with different concentrations (10% and 20%) of PEG solution were compared with the control group. The germination energy of genotype Osk.3.286/1-19 significantly decreased with the 10% PEG treatment, while the 20% PEG concentration significantly reduced germination energy in all genotypes except Srpanjka (Figure 1A). The highest decrease was observed in Tika Taka (23%). However, the percentage of germination for Kraljica was significantly reduced even at the 10% PEG concentration, with a significant decrease in all genotypes at the 20% PEG concentration (Figure 1B). Under the 20% PEG treatment, Kraljica had the highest decrease in germination percentage (17%), whereas Srpanjka’s germination percentage was not significantly affected by either PEG treatment.

To further analyze the effect of different drought stresses on the investigated genotypes, seedling growth traits were recorded. Drought treatment with different concentrations of PEG affected shoot length, with all genotypes showing a significant decrease under both PEG treatments (Figure 1C). The highest decrease in shoot length was observed in Brko and Tika Taka (61% at 20% PEG concentration). The lowest significant reduction was observed in Essekerka, Vulkan, and Srpanjka.

The effect of the lower PEG concentration (10%) on root length was not significant for four genotypes (Super Žitarka, Kraljica, Tika Taka, and Slavonika), while only Vulkan showed a root length that was not significantly different from the control in the 20% PEG solution (Figure 1D). The highest decrease in root length was observed in Brko under the 20% PEG treatment (57%).

**Figure 2:**
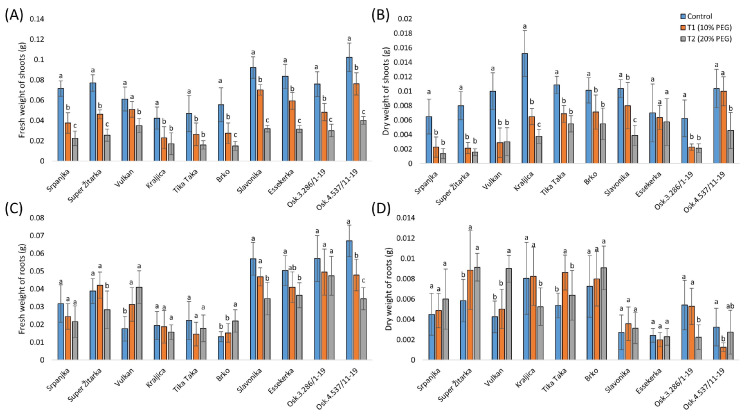
Fresh weight of shoots (**A**), dry weight of shoots (**B**), fresh weight of shoots (**C**), and dry weight of roots (**D**) of ten winter wheat genotypes under control and two polyethylene glycol (PEG) treatments (T1-10% and T2-20%). Data are average values of ten biological replicates ± SD. Significant differences among treatments for each genotype, separately, were assessed by the Fisher LSD test. Trait means with the same letter do not significantly differ at *p* < 0.05.

The effect of a lower concentration (10%) of PEG on the fresh weight of shoots was not significant for Vulkan, while the treatment with 20% PEG significantly reduced the fresh weight of shoots for all genotypes (Figure 2A). The dry weight of shoots in the 10% PEG treatment was not significantly different from the control in Essekerka and Osk.4.537/11-19 (Figure 2B). Under the 20% PEG treatment, the dry weight of shoots was significantly decreased in all genotypes compared with the control, except in Essekerka.

The fresh weight of roots was significantly decreased in the 10% PEG solution for Osk.4.537/11-19, while it was significantly increased in Vulkan (Figure 2C). Under the highest PEG treatment, the fresh weight of roots in four genotypes (Super Žitarka, Slavonika, Essekerka, and Osk.4.537/11-19) was significantly decreased, while it significantly increased in Vulkan and Brko. The effect of the lower concentration of PEG (10%) on the dry weight of roots was not significant for most genotypes, except for Super Žitarka, Tika Taka, and Osk.4.537/11-19 (Figure 2D). Under the highest PEG treatment (20%), the dry weight of roots significantly increased in Super Žitarka and Vulkan and significantly decreased in Kraljica and Osk.3.286/1-19.

### 2.2. Total Metabolite Profiling Under Two Drought Intensities in Wheat

**Figure 3:**
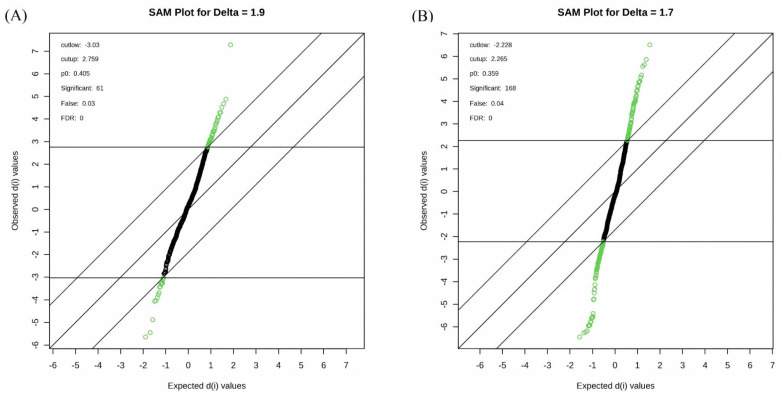
Significant analysis of metabolites (SAM) plot. The green points represent metabolite features that are differentially regulated between control and 10% PEG treatment (**A**) and 20% PEG treatment (**B**). The solid diagonal line represents “observed = expected”. The more the variable deviates from the “observed = expected”, the more likely it is to be significant.

Among the 668 metabolic features analyzed, 54 metabolites exhibited significant changes between the control and the treatment with 10% PEG (*p* < 0.05), and 140 metabolites showed significant changes between the control and the treatment with 20% PEG (*p* < 0.05). These were selected for further analyses (Supplementary Tables S1 and S2). The major groups of metabolites altered due to drought stress induced by the 10% PEG solution included organic acids (12), amino acids (10), carboxylic acids (7), sugars (2), hormones (3), sialic acid (neuraminic acid, N-acetyl), a flavonoid (taxifolin), a disaccharide (turanose MP), a fatty acid (pentacosanoic acid methyl ester), a nucleic acid (uracil), nucleosides (adenosine alpha and inosine), a salt (glycyl-proline), and other organic compounds (12) (Supplementary Table S1, Figure 3A).

**Figure 4:**
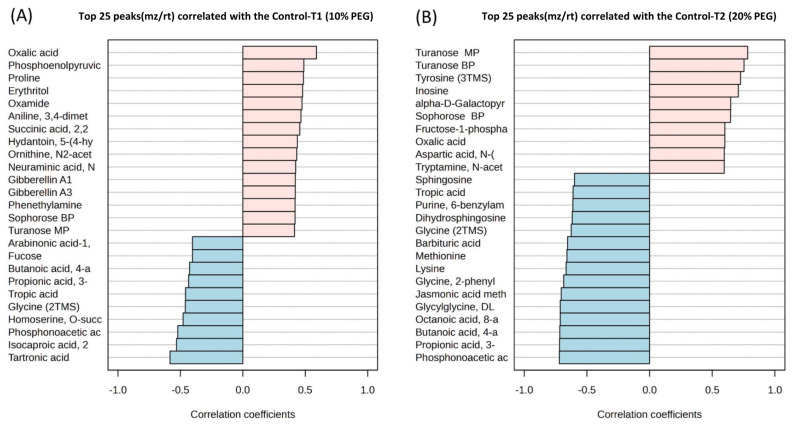
Top 25 metabolites with highest/lowest correlation coefficient from Pearson’s correlation test between control and 10% polyethylene glycol (PEG) treatment (**A**) and control and 20% PEG treatment (**B**). Each row represents the most significant variable identified from the test (*p* < 0.05).

Drought stress induced by 20% PEG treatment altered the content of 20 amino acids and 8 their derivatives or related compounds, 23 organic acids, 20 organic compounds, 16 carboxylic acids, 7 sugars, 6 sugar acids, 5 disaccharides, 4 alkaloids, 4 fatty acids, 2 amino alcohols, 2 monosaccharides, 2 plant metabolites, 2 benzoic acids, a lipid (dihydrosphingosine), a dipeptide (glycylglycine DL), a monoamine alkaloid (beta-D-Fructofuranosyl-(2,1)), a nucleoside (inosine), 3 phenolic acids (homogentisic acid), a carbohydrate (alpha-D-galactopyranosyl-(1,4)-D-galactopyranoside), a nucleotide base (guanine), a hydroxycinnamic acid (ferulic acid trans), a phosphodiesterase family enzyme (myo-Inositol-1-phosphate), a flavonoid (eriodictyol), hormones (gibberellin A3 and zeatin-trans), a salt (glycyl-proline), a phosphoric acid (serine O-phospho), a purine nucleoside (inosine 2-deoxy), an alcohol (ribitol), an acetoic acid (pyruvic acid 3-hydroxy), and a keto acid (pyruvic acid 4-hydroxyphenyl) (Supplementary Table S2, Figure 3B). Figure 4 shows the top 25 highly correlating and significant metabolites (*p* < 0.05) that change with increasing PEG concentration. These include metabolites that increased when PEG was added (shown in red; positive R) or decreased (highlighted in blue; negative R). It was observed that oxalic acid had the highest positive correlation between the control and 10% PEG, while tartronic acid had the highest negative correlation (Figure 4A). The highest positive correlation between the control and 20% PEG treatment was observed for turanose, and the highest negative correlation for phosphonoacetic acid (Figure 4B). The metabolites that were positively correlated between the control and both PEG treatments were turanose, sophorose, and oxalic acid. Tropic acid, glycine, butanoic acid, propionic acid, and phosphonoacetic acid were the most negatively correlated in the control and PEG treatments.

**Figure 5:**
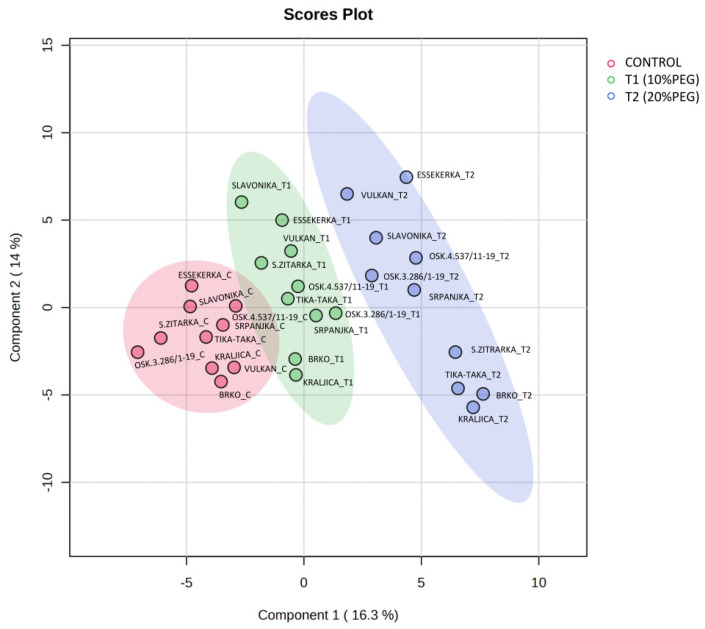
Partial least square discriminant analysis (PLS-DA) in seedlings of ten wheat genotypes under control and two drought conditions (T1 and T2). Samples at control and drought treatments did not overlap with each other indicating an altered state of metabolite levels in the wheat seedlings.

Subjecting the metabolite data to a PLS-DA revealed high variability in metabolite contributions to drought responses (Figure 5). PC1 explained 35.9% of the variability, while PC2 explained 9.6%. All control samples tended to cluster together, indicating similar metabolite profiles. Additionally, samples grown under severe drought conditions (20% PEG) clustered separately from the control samples. Samples grown under mild drought stress (10% PEG) were located in the intermediate region between control and severe drought, suggesting that mild drought stress represents a transition state. The PLS-DA biplot revealed that Vulkan in T2 was located on the opposite side of Vulkan in the control, indicating a direct negative relationship between the metabolites of these two treatments in this genotype. However, Srpanjka showed the least changes in metabolite profile across the three treatments, followed by Osk.4.537/11-19, which was closest to the origin. A group in the lower right quadrant consisted of four genotypes from the 20% PEG treatment (Super Žitarka, Brko, Tika Taka, and Kraljica) and six genotypes (Vulkan, Slavonika, Essekerka, Osk.3.286/1-19, Osk.4.537/11-19, and Srpanjka) in the upper right quadrant, which positively contributed to PC1. The group with the 10% PEG treatment (T1) that positively contributed to PC1 consisted of four genotypes (Srpanjka, Osk.3.286/1-19, Brko, and Kraljica) in the lower right quadrant.

Positive contributions to PC2 were observed in six genotypes under the 20% PEG treatment (Vulkan, Slavonika, Essekerka, Osk.3.286/1-19, Osk.4.537/11-19, and Srpanjka) in the upper right quadrant and a group of six genotypes (Slavonika, Essekerka, Super Žitarka, Vulkan, Tika Taka, and Osk.4.537/11-19) in the upper left quadrant under the T1 drought treatment, along with one genotype (Essekerka) under the control treatment.

## 3. Discussion

New plant growth is greatly influenced by successful germination and seedling development. After seed germination, when the radicle protrudes, it is followed by the post-germinative growth of the seedling [21]. Furthermore, most of the future growth of a seedling depends on the cell divisions that occur in both the root and shoot meristems within the mature plant embryo [22]. At the germination and seedling stages, drought causes various physiological and biochemical changes, leading to dehydration of plant protoplasm and metabolic disorders, which affect normal plant growth [23]. Our current knowledge is limited in understanding the contrasting metabolic changes in wheat seedlings and the relationship between altered metabolite levels and performance under drought stress.

### 3.1. Morphological Changes in Wheat Seedlings from This Study

Previous research has shown that drought impairs germination and leads to poor stand establishment [24,25,26]. Although many seeds may remain viable, unfavorable seedling growth might occur. Previous studies report that germination, root, and leaf growth of wheat genotypes are considerably influenced by drought stress, depending on the drought tolerance of the genotypes [27]. The current results show that drought stress at 20% PEG has a strong inhibitory effect on the germination energy and percentage of germination in most genotypes. The reduction in germination with increased PEG levels could be associated with high seed nutrient imbalance, toxic ions, and reduced soluble osmotic potential [28]. Similar findings were recorded in rice genotypes, showing that the germination percentage, germination energy, germination rate, and germination index decreased as PEG concentrations increased [29]. Additionally, previous research by Duvnjak et al. [26] observed that PEG treatments negatively affected germination energy in all wheat varieties in a dose-dependent manner. However, in the current research, the genotype Srpanjka did not show significant changes in germination energy and percentage of germination under both drought treatments. Even higher reductions in wheat germination, up to 54% under PEG treatment, indicating that some genotypes better tolerated increasing osmotic potentials.

Drought differentially affects various plant parts, such as shoot and root length. For example, early seedling growth in rice subjected to drought stress results in a significant reduction in shoot and root length [30]. In the current study, the shoot length of ten genotypes was significantly reduced with increased PEG-induced drought levels. Similar findings, who showed that shoot length was reduced in wheat seedlings under PEG treatment by up to 54%. Under severe water deficiency, cell elongation in higher plants can be inhibited by the interruption of water flow from the xylem to the surrounding elongating cells [31]. Furthermore, Kerbauy [32] concluded that under drought stress conditions, seedling growth is affected due to reduced water uptake by the plants and lower cell turgor pressure. At the root level, drought reduces root length, density, and dry weight, which decreases water uptake capacity [16]. However, in the current research, it was evident that root growth appears to be less affected by milder drought than shoot growth. It should also be noted that root length is negatively correlated with shoot length and relative water content (RWC) [33], which could mean that a larger decrease in PEG treatments would result in higher RWC.

An unsolicited effect of drought on plants is the reduction in fresh and dry biomass [34]. In our study, the fresh and dry weights of shoots were significantly reduced in all genotypes due to 10% and 20% PEG stress treatments. However, the fresh and dry weights of roots indicated that slight drought stress increased these traits in most of the genotypes. Duvnjak et al. [26] revealed that the dry weight of roots in wheat seedlings was negatively correlated with germination energy, shoot length, fresh weight, and salicylic acid. Only the genotype Srpanjka showed no significant changes in the fresh and dry weights of roots. Under drought, plants may try to adapt to stress by altering their morphological structure, physiological, and biochemical functions to ensure the development of plant organs. As a result, leaf area may be reduced while root activity increases [27].

### 3.2. Metabolic Changes in Two Drought Intensities

Metabolomics is an effective tool for testing plant responses to different stresses, but the relationship between metabolites and their functions in response to drought stress remains uncertain. The study by Zhang et al. [35] demonstrated that drought stress increased levels of various small carbohydrates and soluble amino acids in the leaf, stem, root collar, and root. Different amino acids, as well as intermediates of the Krebs cycle and glycolysis, showed a reduction at the whole-plant level due to drought stress. In the current study, the 54 and 140 differential metabolites identified in the control and 10% PEG treatment and in the control and 20% PEG treatment, respectively, were clustered into carboxylic acids, amino acids, sugars, organic compounds, hormones, flavonoids, fatty acids, nucleic acids, phenolic acids, salts, nucleosides, cytokinins, and other compounds based on their comprehensive functions/pathways (Figure 3A,B). In the research by Guo et al. [18], mass spectrometry analysis under drought treatment enabled the identification of 56 metabolites, including amino acids, organic acids, carbohydrates, alkaloids, flavones, purines, and pyrimidines. Another study documented changes in soluble sugars, amino acids (especially proline), soluble proteins, betaine, and organic acids under drought stress [36]. Proline was the amino acid that significantly changed under the 10% PEG treatment in this study.

Metabolites can protect plants by exercising various physiological responses, such as strengthening membrane integrity, harmonizing enzymatic/antioxidant activity, and fulfilling water requirements [37]. Guo et al. [18] indicated that phenolics are important antioxidants that plants use to avoid oxidative damage caused by drought stress. Plants maintain turgor pressure and cell volume during drought through osmotic adjustment, accumulating and integrating compatible solutes like proline, sugars, and free amino acids to keep metabolic functions active [38]. More drought-tolerant wheat genotypes enhance physiological responses by upregulating regulatory genes and producing more sugars, organic acids, and important amino acids in shoots to maintain growth under drought stress [19]. Furthermore, plants can activate many enzymes, such as phenylalanine ammonia lyase, chalcone synthase, and phosphoenolpyruvate carboxylase, suggesting a shift from sucrose production to adaptation to drought [39]. In this experiment, phosphoenolpyruvic acid had one of the highest peaks in the 10% PEG treatment, indicating enhanced carboxylation of phosphoenolpyruvate to form oxaloacetate under mild stress. The metabolites of the control and two drought stresses were separated into three groups using PLS-DA. Among the 25 differential metabolites that showed the highest correlation coefficient between control and drought, three metabolites were upregulated by drought stress: oxalic acid, sophorose, and turanose. Four metabolites decreased under both drought treatments: propionic acid, tropic acid, glycine, and phosphonoacetic acid.

#### 3.2.1. Organic Acids and Compounds

Organic acids play important roles in photosynthesis, nutrient uptake, tricarboxylic acid cycle metabolism, and drought tolerance in plants [40]. They are primarily formed in plants through the Krebs cycle, glyoxylate cycle, and photosynthesis. Moreover, organic acids such as succinic acid, malic acid, galacturonic acid, citric acid, glyceric acid, isocitric acid, and caffeic acid are involved in the plant response to drought [41]. Our study revealed that succinic and aspartic acids notably increased the plant response to mild and severe drought stress, respectively. In the study by Khan et al. [42], succinic acid, malic acid, and galacturonic acid also played roles in the response to long-term drought stress. It was also demonstrated that aspartic acid has an important role in plant resistance to drought stress [43].

Khosravi-Nejad et al. [44] showed that drought stress in wheat decreased malic acid, pyruvic acid, and citric acid content. Conversely, in our experiments, malic acid increased under both drought intensities compared with the control. It was also previously reported that malic acid increases under drought conditions, enhancing plant tolerance to water deficit by modulating osmotic potential [45]. Similarly, malic acid is the main organic acid secreted in maize roots, solubilizing phosphate for plant utilization [46]. The highest decrease under mild drought stress in this study was observed for arabinonic acid, and barbituric acid in the case of severe drought stress. Barbituric acid forms C-linked glycosides with many sugars [47], which could be an adaptation and response of plants to stress.

#### 3.2.2. Amino Acids, Hormones, and Benzenoids

Amino acids are essential components of proteins and are involved in a wide variety of biochemical and physiological processes [48]. They play a crucial role in the biosynthesis of proteins, serve as building blocks for numerous biosynthetic pathways, act as precursors for various specialized metabolites, and are important in signaling processes in response to stress [49]. Increased amino acid biosynthetic capability and ROS scavenging ability, resulting from higher antioxidant activities and increased flavonoids, may be mechanisms underlying drought tolerance [36].

Increased proline in wheat plants helps overcome drought stress by supplying energy for growth and survival [50]. Rahman et al. [51] showed that amino acids increased under drought stress in wheat at the post-anthesis stage. In the current research, proline increased in drought-treated plants compared with the control, possibly to stabilize DNA, membranes, and protein complexes and provide carbon and nitrogen as an energy source to combat drought stress [52].

Sharma et al. [53] reported that drought stress decreases photosynthesis efficiency, increases ROS production and oxidative damage, increases abscisic acid accumulation and proline production, enhances antioxidant enzyme activity, and decreases cytokinin accumulation. In contrast, our research revealed that trans-zeatin was significantly elevated in both PEG treatments. The production of trans-zeatin is important for root and shoot meristematic activity, as it acts as a hormonal signaling transporter around its biosynthetic sites, playing a role in plant growth and drought response regulation [54]. Phenylalanine, tryptophan, and tyrosine are considered central molecules in plant metabolism, especially under environmental stresses [42]. Tryptophan significantly increased under both mild and severe drought during our experiment, while tyrosine and acetyl-5-hydroxy-tryptamine increased only under severe drought. Significant increases in proline, methionine, serine, asparagine, phenylalanine, aspartic acid, GABA, glycine, 5-hydroxynorvaline, threonine, and valine have been previously reported under drought [42]. Gibberellins are hormones that affect plant growth and development during germination and flowering, including seed germination [55]. In wheat, gibberellins play a crucial role in controlling and promoting germination [56]. From the current research, it can be suggested that under 20% PEG stress, the production of gibberellin A3 is inhibited, indicating that stronger drought stress is more rate-limiting in bioactive gibberellin biosynthesis. Gibberellins A1 and A3 were detected in germinating wheat grains under normal conditions [57,58]. It has been suggested that gibberellin content and metabolism are positively correlated with faster shoot growth rates in wheat hybrids or heterosis [59].

Two benzoic acids, salicylic and vanillic acid, were downregulated in the 20% PEG treatment. In some studies, vanillic acid increased due to abiotic stresses, including drought [60]. Salicylic acid plays a role in regulating important plant physiological processes, such as photosynthesis, nitrogen and proline metabolism, glycinebetaine formation, the antioxidant defense system, and plant–water relations under stress conditions [61].

#### 3.2.3. Sugars, Amino Sugars, and Carboxylic Acids

Sugars play a crucial role in plant growth and development, influencing all stages of the plant’s life cycle and interacting with other signaling molecules, including phytohormones [62]. In control and PEG treatments, turanose levels increased, with even higher levels reached during severe drought. Turanose is a disaccharide present naturally in plants and is implicated in altering plant physiology via the hormones ethylene and auxin [63]. In addition, turanose levels drastically increased in wheat accessions infected with the fungus *Tilletia caries* [64]. Increased levels of sugar help stress tolerance by increasing the osmotic potential in the plant [65]. The accumulation of many sugars, for example, fructan, trehalose, galactinol, and sugar alcohols such as mannitol and D-inositol, also plays a role in drought tolerance [42]. However, the type of sugars that accumulate vary depending on species, organ, and developmental stage. During mild drought stress, *N*-acylneuraminic acid significantly increased compared with controls. *N*-acetylneuraminic acid is abundant in terminal modifications of protein-linked glycans. It was demonstrated that *N*-acetylneuraminic acid was detected in tobacco cells, mung bean sprouts, apples, and bananas [66]. The concentration of total carbohydrates is limited under drought conditions [52]. Under 20% PEG stress, only alpha-D-galactopyranosyl-(1,4)-D-galactopyranoside was positively correlated with the control.

Regarding carboxylic acids, oxalic acid showed the highest positive correlation in the 10% PEG treatment among other metabolites and was also significant in the 20% PEG treatment. High concentrations of oxalic acid are known to induce programmed cell death, while low concentrations increase plant resistance to fungi [67]. Additionally, oxalic acid converts inorganic phosphorus to plant-available P in soil solution, enhancing P uptake [68]. Oxalates are involved in seed germination, calcium storage and regulation, ion balance, detoxification, structural strength, and insect repulsion in plants [69]. In the current study, it was evident that oxalic acid increased in seedling tissue under drought stress. This likely led to increased germin mRNA and oxalate oxidase activity, as previous research has shown that this occurs under both biotic and abiotic stresses [70].

#### 3.2.4. Fatty Acids

Lipid content varies in plants when exposed to various stresses. These conditions induce fatty acid saturation, increasing membrane rigidity [19]. Fatty acids play specific roles in signaling events and metabolic processes [71]. 8-aminooctanoic acid, octadecanoic acid, and 2-oxovaleric acid showed negative correlations between the control and 20% PEG treatment. Maintaining high levels of fatty acids is important, as desaturation enables plants to stabilize membrane fluidity and reduce membrane damage caused by drought [72]. Gigon et al. [73] also reported an increase in unsaturated fatty acid levels during drought stress in *Arabidopsis thaliana*. Along with other metabolites, unsaturated fatty acids are crucial for drought tolerance in thyme [74]. Among the top 25 metabolites that correlated between the control and 20% PEG treatment, only 8-aminooctanoic acid was represented. This aligns with the research by Khosravi-Nejad et al. [44], which showed that organic acids exhibited the most variation in metabolic profiling in wheat, while fatty acids showed minimal change during the treatments. Previously, octanoic acid was also detected in disease-stressed shoots of wheat seedlings [75].

## 4. Materials and Methods

### 4.1. Wheat Material

Seeds of 10 winter wheat genotypes supplied by Agricultural Institute Osijek were used for this study (Table 1). Descriptions of recognized wheat varieties may be found in catalogs at https://www.poljinos.hr/katalozi/ (accessed on 3 October 2024).

### 4.2. Experimental Layout

The experiment was conducted under controlled conditions in a plant growth chamber (Aralab, Rio de Mouro, Portugal) and designed as a completely randomized block design with eight replicates for each genotype per treatment. Prior to germination, wheat seeds were sterilized with 70% ethanol for 1 min and washed twice in deionized water (dH_2_O). Germination assays were performed by evenly distributing 40 seeds in glass jars (H 9 × W 10 cm, V = 0.5 L) lined with filter paper. Sterile deionized water (10 mL) was added daily to the control jars, while the desired amounts of osmotic solutions were added to mimic drought stress. Seeds were germinated at 25/20 ± 2 °C day/night, with a relative humidity of 60% and a photoperiod of 16 h for 7 days. Polyethylene glycol (PEG) was added to the water at concentrations of 10% (treatment 1; T1) and 20% (treatment 2; T2) to reduce the water potential of the solution, thereby inducing drought stress in the plants. In our experiments, PEG-6000 (Sigma-Aldrich, Darmstadt, Germany) was used without causing toxicity to cells [23]. Seeds were checked regularly for germination and were considered germinated if the emerged radicles measured approximately 2 mm [76]. After 7 days of growth, the following germination attributes and morphological parameters were measured. For metabolomics analysis, the tissue of the seedlings was lyophilized.

### 4.3. Morphological Traits

To investigate the effects of drought stress, six morphological traits (germination energy, percentage of germination, shoot and root length, and fresh and dry weight of shoots and roots) were measured using seedling samples collected after seven days of drought stress induced by two PEG treatments. Germination energy was calculated in eight replicates by the formula: (number of seeds germinated on the fourth day/total number of seeds) × 100. The percentage of germination was calculated in eight replicates by the following formula: (number of seeds germinated on the seventh day/total number of seeds) × 100. Shoot and root lengths (mm) were measured in eight replicates using a ruler on the seventh day of the experiment. Fresh and dry weights of shoots and roots were obtained in eight replicates by weighing them separately, and again after 24 h of drying in a lab dryer at 105 °C. Shoot and root biomass was expressed in terms of fresh weight (FW) and dry weight (DW) per plant.

### 4.4. Metabolic Profiling

Prior to extraction for metabolic analyses, the seedlings were flash frozen in liquid nitrogen and ground in 10 mL plastic tubes together with a grinding ball for 2 min per sample in automatic Labman’s cryogenic grinding system (Labman, Middlesbrough, UK). Polar and semipolar metabolites were extracted from 15 mg of deep-frozen homogenized plant material using polar metabolite extraction protocol according to Riewe et al. [77]. Extraction was proceeded by adding 1 mL of chilled extraction buffer (2.5:1:1 *v*/*v* MeOH/CHCl_3_/H_2_O) containing 1 μL of a 2 mg/mL stock solution of ^13^C-sorbitol, and D4-alanine to the flash-frozen and pulverized tissue. Following 15 min incubation at 4 °C, 0.400 mL H_2_O was added and centrifugated for 15 min at 4 °C and Vmax. Extraction was split into three batches and aliquots of 50 μL of polar phase into GC glass vials. Additionally, GC vials containing aliquoted samples were placed in a Speedvac overnight, crimped, and stored in sealed plastic bags with silica gel at −80 °C until analysis. Dried extracts were in-line derivatized directly prior to injection [78] using a Gerstel MPS2-XL autosampler (Gerstel, Mühlheim/Ruhr, Germany) with the front inlet temperature set at 200 °C, analyzed in splitless mode, using a LECO Pegasus BT time-of-flight mass spectrometer (LECO, St. Joseph, MI, USA, https://www.lecosoftware.com/chromatof, accessed on 10 March 2024) connected to an Agilent 8890 gas chromatograph and helium as the carrier gas at 1.0 mL min^−1^ flow and linear velocity as flow control mode. The capillary column used was an Agilent DB-35MS (30 m × 0.25 mm × 0.25 μm). Fatty acid methyl esters (FAME MIX: C9-C31) were used in the determination of the retention time index [79].

Metabolic features were identified by employing the LECO ChromaTOF software in conjunction with the Golm Metabolome Database (GMD, http://gmd.mpimp-golm.mpg.de/, accessed on 11 March 2024). Peak intensities were determined using the TargetSearch package [80] within the R software (4.3.1 version, R Core Team, 2023). For normalization, the content of the internal standards D4-alanine and ^13^C Sorbitol were utilized. All metabolic class identifications were manually validated based on GOLM, KEGG (https://www.genome.jp/kegg/pathway.html, accessed on 13 March 2024), and HMDB (https://www.hmdb.ca/, accessed on 13 March 2024) metabolites databases.

### 4.5. Statistical Analysis

Samples were collected from each jar, and after data collection, they were tested for normality, which indicated that the data were normally distributed. Morphological parameters were expressed as the mean value of eight replicates ± standard deviation. Differences among treatments for each genotype were assessed by a one-way analysis of variance (ANOVA), followed by the Fisher test using Statistica software (version 14). Morphological parameters were analyzed as dependent variables, with genotype and treatment as fixed effects in a one-way ANOVA.

Partial least squares discriminant analysis (PLS-DA) and significant analysis of metabolites (SAM) were performed using MetaboAnalyst 6.0.

## 5. Conclusions

The PEG solution inhibited seed germination, but the percentage of germination remained high, indicating that all plant seeds had a certain level of drought tolerance, with Srpanjka showing the highest tolerance. There was a decrease in shoot and root-related traits with the rise in osmotic potential levels. Under mild stress, the highest negative correlation with the control was observed for organic acids, compounds, and carboxylic acids. However, under severe drought stress, the contents of carboxylic acids, organic compounds, and some amino acids showed the highest negative correlation with the control, indicating damage to the wheat seedlings. Among the top 25 metabolites that showed a positive correlation between the control and both PEG treatments were oxalic acid, sophorose, and turanose. In contrast, negative correlations were observed for butanoic acid, 3-ureidopropionic acid, tropic acid, glycine, and phosphoenolpyruvic acid. The drought tolerance of wheat genotypes is very complex, and further research is needed to explore the mechanisms of wheat seedlings’ response to drought. By identifying and quantifying key metabolites, researchers can better understand the metabolic status of an organism and develop targeted interventions for various conditions.

## Figures and Tables

**Table 1:** Origin and registration year of ten investigated wheat genotypes.

No.	Wheat Genotype	Origin	Registration Year
1	Srpanjka	AIO *, Croatia	1989
2	Super Žitarka	AIO, Croatia	1997
3	Vulkan	AIO, Croatia	2009
4	Kraljica	AIO, Croatia	2010
5	Tika Taka	AIO, Croatia	2014
6	Brko	AIO, Croatia	2020
7	Slavonika	AIO, Croatia	2023
8	Essekerka	AIO, Croatia	2023
9	Osk.3.286/1-19	AIO, Croatia	-
10	Osk.4.537/11-19	AIO, Croatia	-

* AIO—Agricultural Institute Osijek.

## Data Availability

The data that support the findings are available on request from the corresponding author.

## Supplementary Materials

The following supporting information can be downloaded at https://www.mdpi.com/article/10.3390/plants14010010/s1. Table S1: Sam signatures between control and 10% PEG treatment; Table S2: Sam signatures between control and 20% PEG treatment.
